# Long-term evaluation of hard and soft tissues around transmucosal implants with a convergent neck: a 10-year cohort study

**DOI:** 10.1007/s10006-026-01536-6

**Published:** 2026-03-09

**Authors:** Carlo Prati, Andrea Spinelli, Jacopo Lenzi, Maria Giovanna Gandolfi, Achille Tarsitano, Giovanni Badiali, Fausto Zamparini

**Affiliations:** 1 Endodontic Clinical Section, Dental School, DIBINEM, University of Bologna, Bologna, Italy; 2 DIBINEM, University of Bologna, Bologna, Italy; 3 Laboratory of Green Biomaterials and Oral Pathology, Dental School, DIBINEM, University of Bologna, Bologna, Italy; 4 Oral and Maxillo-Facial Surgery Unit, DIBINEM, University of Bologna, Bologna, Italy; 5 Endodontic clinical section, Dental School, Department of Biomedical and Neuromotor Sciences, University of Bologna, Bologna, 40125 Italy

**Keywords:** Dental implants, MBL, PES, Transmucosal implants, Prospective study

## Abstract

**Purpose:**

The aim of the present study was to analyse the 10-year hard and soft tissue variations around two-piece transmucosal implants characterized by a hyperbolic neck.

**Methods:**

This cohort study was initiated in 2014 and included patients receiving single-tooth implants as part of routine clinical care. A flapless approach was followed to place implants in healed crests (ITI bone 2 or 4). A post-extractive placement was performed in presence of unrestorable teeth without acute infections (ITI bone 1). Implant loading procedures were similar in all cases, following a provisional crown after 3 months and a definitive metal ceramic restoration at 4 months. Patients were enrolled in a structured recall program with clinical and radiographic evaluations. Marginal Bone level (MBL) and Pink Esthetic Score (PES) were used as indexes of hard and soft tissue stability. The onset of new systemic diseases and pharmacological therapies were recorded at 6- and 10-year follow-up. MBL and PES were modeled as continuous outcomes using linear regression with cluster-robust standard errors at the patient level. Separate models were fitted including each predictor (age group, CVDs, antihypertensive therapy), follow-up time (six vs. ten years), and their interaction.

**Results:**

After 10 years, 37 implants in 30 patients were available for clinical or radiographic evaluation. Implant survival and success rates were 100% at the 10-year follow-up, with marginal bone loss remaining below 2 mm. At 10 years, mean MBL was 0.90 (95% CI 0.68 to 1.12 mm) while mean PES was 11.1 (95% CI, 10.6 to 11.6). No statistically significant associations were observed between cardiovascular conditions or antihypertensive therapy and changes in MBL (*p* > 0.050); in contrast, PES soft tissue scores demonstrated a modest but significant reduction between six and ten years (mean change − 0.8; *p* < 0.001), with consistently lower values in older patients and in patients with cardiovascular disease.

**Conclusions:**

Within the limitations of this study, transmucosal implants with a convergent neck showed stable marginal bone levels over a 10-year follow-up. The occurrence of new systemic conditions related to aging did not appear to significantly affect MBL in this cohort. However, their potential impact should be further investigated in future long-term studies.

## Introduction

A follow-up of ten years or longer is important to properly evaluate the outcome of dental implants and their associated surgical and prosthetic protocols. Some clinical studies investigated tissue-level and transmucosal implants, but evidence is currently limited to a small number of selected implant designs [1–6]. Current ten-year follow-up studies reported high implant survival rates (95–99%) and success rates ranging from 92% to 97% [4–6].

Approximately a decade ago, a transmucosal two-piece implant design characterized by a convergent (hyperbolic) neck and an implant platform positioned 0.5–1.0 mm above the mucosal surface was introduced as an innovative approach [7, 8]. This design aimed to eliminate the need for a second-stage surgical re-entry for cover screw exposure and to reduce biological and biomechanical stress on the peri-implant soft tissue tunnel [7–11]. The morphology of soft tissues around exposed neck/collar were stabilized by conservative approach and preserved gingival tissues [12]. Subsequent clinical studies demonstrated satisfactory outcomes up to six years after implant placement with low marginal bone loss (MBL) and favorable soft-tissue parameters [8–11]. Long-term (10-year) data for this implant design are currently lacking.

MBL is one of the main measures of peri implant bone stability and quality during time. Other indexes, such as Pink Esthetic score (PES), were conceived to assess the soft tissue stability around implants [13]. Taken together, it is possible to analyze hard and soft tissue stability around implant rehabilitations.

The aim of the present study was to analyse the soft tissue modifications and marginal bone loss of hyperbolic neck implants placed at least ten years before, with specific focus on the interval between six and ten years and on the potential impact of newly occurring systemic conditions during long-term follow-up. All patients included were enrolled in a recall program with annual hygiene appointments and radiographic evaluations.

## Materials and methods

### Study setting and patient selection

This prospective longitudinal cohort study started in 2014. The study was conducted as part of standard clinical care in accordance with institutional and local regulations in place at the time of patient recruitment. All surgical and prosthetic procedures were performed as part of routine clinical care using commercially available dental implants, with no experimental devices, materials, or deviations from standard treatment protocols.

At the time of study initiation, formal approval by an institutional ethics committee was not required for studies involving standard-of-care treatments at the study center, in accordance with local regulations and institutional practice. All procedures were conducted in compliance with the principles of the Declaration of Helsinki [14].

Prior to participation, all patients provided written informed consent for the surgical and prosthetic procedures and consented to the use of their clinical and radiographic data for research purposes. Patient confidentiality was strictly maintained throughout the study. The manuscript was prepared in accordance with the STROBE guidelines for cohort studies [15] and complied with the recommendations of Dodson [16]. Patients were eligible to participate when fulfilling to the following criteria.

### Inclusion criteria

- Age 18–75.
- Presence of a single failing tooth or a single-tooth edentulous space with adjacent teeth present.
- Willingness to participate in hygiene recall and implant monitoring.
- Smoking less than 10 cigarettes per day.

## Exclusion criteria

- ASA score ≥ 3 or other systemic contraindications to surgery.
- Poor oral hygiene or lack of motivation.
- Active periodontal disease (pocket depth > 4 mm and bleeding on probing).
- Uncontrolled diabetes or other systemic conditions impairing healing or osseointegration.
- Alcohol or drug abuse.
- Pregnancy or lactation.
- Malocclusion or occlusal dysfunction (e.g. bruxism).
- Previous bisphosphonate therapy.

## Patient allocation

Implant timing was determined according to the ITI Consensus Conference classification [17]: **Immediate placement (Type 1)**: Implant placed into a fresh extraction socket after removal of teeth affected by chronic periapical pathology. Lesions were identified via periapical radiolucency. **Early placement (Type 2)**: Implant placed in partially healed bone 4–8 weeks after extraction of teeth with acute periapical lesions, abscesses, or clinical symptoms. **Delayed placement (Type 4)**: Implant placed in mature edentulous bone 10–12 months post-extraction. All cases followed strict criteria aimed at achieving optimal clinical outcomes (judgmental allocation) [18].

## Pre-surgical protocol

One day before surgery, patients underwent antibiotic treatment with: Amoxicillin/clavulanic acid 1 g (Augmentin, GSK, UK), 12 h prior to surgery and Chlorhexidine digluconate 0.12% gel (Corsodyl Gel, GSK, UK), applied 3 times daily.

## Implant surgery

The surgical protocol has been fully described in previous investigations [11, 19]. In brief, all surgeries were performed under local anesthesia with 30 mg/mL mepivacaine (Carboplyina, Dentsply Italia, Rome, Italy), under sterile conditions. One-stage approach was used in all cases. No surgical guides were utilized.

### Immediate implant placement

After extraction, sockets were inspected and prepared using a 1.2 mm drill, following the palatal bony wall as a guide. Twist and calibrated drills (225 rpm) were used to achieve apical anchorage at least 3 mm beyond the root apex. Implants (Prama, Sweden & Martina, Italy), featuring a 2.8 mm anodized convergent neck and ZirTi surface, were inserted so the rough surface aligned with the bone crest and the smooth neck emerged 1–1.5 mm above the gingiva. A 1.0 mm cover screw was placed and maintained throughout healing. A surgical dressing (Coe Pack) was applied for 3–6 days.

### Early and delayed placement

A flapless procedure was performed by an experienced operator. Site preparation involved marking with a 1.2 mm drill and subsequent drilling through mucosa, cortical, and cancellous bone. The implants were inserted transmucosal, positioning the smooth neck 0.5–1.0 mm above the gingiva. A cover screw (1.0 mm) was applied; no sutures were required.

### Provisional restoration with adhesive bridge

When requested, an interim adhesive bridge was placed during healing. Palatal/lingual enamel of adjacent teeth was etched with phosphoric acid (3 M ESPE), rinsed, and bonded using Scotchbond Universal. Relyx Ultimate resin cement was used to secure the provisional bridge.

### Prosthetic rehabilitation

In all cases, impressions were taken approx. 3 months post-implantation using a polyether material (Permadyne/Garant, 3 M ESPE) in custom trays (pick-up technique). Custom titanium abutments were designed with the abutment-implant junction positioned 0.5–1.0 mm above the soft tissues. Temporary resin crowns were cemented with TempBond (Kerr, USA), avoiding gingival compression. The definitive metal-ceramic crown was delivered 3–4 weeks later, cemented with polycarboxylate cement (Heraeus Kulzer GmbH, Germany). Great care was taken to remove any cement residue. All prosthetic procedures were performed by two experienced prosthodontists. Supportive periodontal care, including hygiene recall, scaling and root planing, and oral hygiene instruction, was provided every 6–12 months during the first 3 years. Thereafter, patients were enrolled in an annual recall program.

### Follow up, hard and soft tissue evaluations

Intraoral periapical radiographs were carried out using a paralleling technique with Rinn-holders [19]. A careful standardization was carried out before patients enrollment. The following parameters were used: target-film distance was approx. 30 cm, 0.41 s exposure time,70 kV voltage and 8 mA intensity. X-rays development was performed in a developed unit and according with manufacturer instructions (Euronda s.p.a., Vicenza, Italy): standard room temperature (25 °C) with 12 s developing and 25s fixing time. When not fulfilling these parameters, patients were asked to make another radiograph. Periapical radiographs have been evaluated as previously reported [11, 19]. All X-rays were scanned with a slide scanner with a resolution of 968 dpi and a magnification factor of x20. The known length and diameter of implants were used to calibrate the measurement.

The crestal marginal bone and the bone-implant interface were examined to evaluate the marginal bone morphology. MBL was assessed at the mesial and distal implant surfaces by measuring the distance between the reference point of the implant platform to the most coronal bone-to-implant contact level using a scale divided into 0.1 mm steps and corrected according to the known height and width of each implant.

Radiographic evaluation was performed in single-blind by one additional examiner. Before evaluating the radiographs, the examiner was calibrated by using well-defined instructions and reference radiographs with different marginal bone level measures.

For the present investigation we analysed the 10-year follow up hard and soft tissue success, survival rates, drop-out, and occurrence of new systemic diseases and new medical therapies. Analyses of longitudinal changes in MBL and PES were restricted to implants with available clinical or radiographic data at the six-year follow-up and re-evaluated at ten years.

Based on the following criteria, implant success at the 10-year follow-up was determined: no bleeding on probing, absence of suppuration, and a maximum bone loss of ≤ 2 mm (accounting for an expected loss of 1 mm in the first year, followed by an annual loss not exceeding 0.1 mm). Periimplantitis was diagnosed in cases where bleeding on probing or suppuration was present, the probing depth was ≥ 6 mm, and bone loss exceeded 2 mm [20, 21].

Systemic diseases and new medication regimens were recorded and updated during the hygienic recall appointments.

### Statistical analysis

Baseline characteristics were summarized as counts and percentages. For longitudinal analyses, implants were analyzed with repeated observations at 72 months (six years) and 120 months (ten years). MBL and PES were modeled as continuous outcomes using linear regression with cluster-robust standard errors at the patient level to account for multiple implants per subject. Separate models were fitted including each predictor (age group, CVDs, antihypertensive therapy), follow-up time (six vs. ten years), and their interaction. Contrasts were estimated to quantify between-group differences at each follow-up, within-group changes over time, and interaction terms. Other systemic conditions and therapies were recorded but were too infrequent to allow meaningful inferential analyses. The analytical sample therefore represents implants with complete follow-up information at both six and ten years. All analyses were conducted using Stata 18 (StataCorp. 2023. *Stata Statistical Software: Release 18*. College Station, TX: StataCorp LLC). Two-sided *p*-values ≤ 0.05 were considered statistically significant.

## Results

### Patients characteristics at end-line (10 years)

**Fig. 1:**
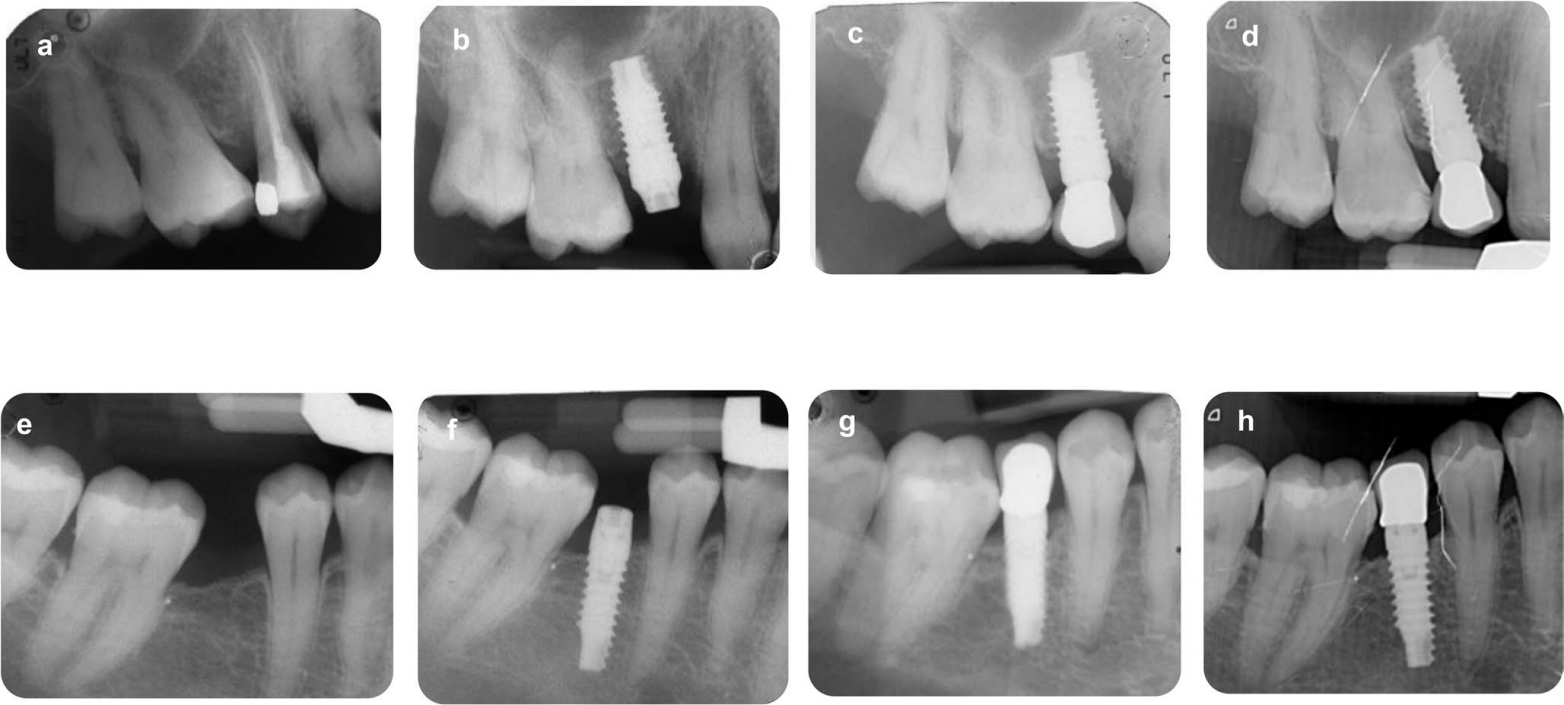
Some representative cases treated in 2014–2015. Immediate post extractive case (**a**, **b**) and delayed implant insertion case (ITI 4 bone) (**e**, **f**). In both cases, loading procedures started 3 months after insertion. Please note that the crown ended in correspondence of the hyperbolic/convergent neck. initial MBL was observed during the first 3 years of load (**c** and **g**). At 10 years (**d** and **h**), MBL resulted stable and no complications were observed

Of the 67 implants placed in 48 patients at baseline, 14 patients (*n* = 22 implants) dropped out due to geographical location. Eight implants were not evaluable due to patients deaths (*n* = 4), leaving 37 implants in 30 patients with clinical or radiographic data at ten years. The initial age of the 48 patients at time 0 was approx. 57.0 years. Two thirds of the cohort were younger than 60 years (67%) when implants were placed, just over half were female (53%), and the large majority were non-smokers (90%). Table 1 summarizes the baseline characteristics and the occurrence of new systemic conditions and drug therapies among these patients. Figure 1 reports 2 representative cases of implant treated in 2014–2015 and followed for 10 years.

**Table 1:** Baseline Characteristics and Occurrence of New Systemic Conditions and Drug Therapies in Patients With at Least One Evaluable Implant at Ten Years

	*n*	%
Age Group, y		
<60	20	67
≥60	10	33
Gender		
Male	14	47
Female	16	53
Smoking Habits		
No	27	90
Yes	3	10
New Medical Systemic Conditions		
Cardiovascular diseases	8	27
Oncological diseases	1	3
Endocrine disorders	1	3
Autoimmune disorders	1	3
Depression	1	3
Metabolic disorders	1	3
Gastrointestinal disorders	1	3
Number of New Medical Conditions		
0	18	60
1	10	33
2	2	7
New Drug Therapies		
Antihypertensive agents*	10	33
Corticosteroids	3	10
Proton pump inhibitors	3	10
Immunological/immunosuppressive agents	2	7
Anticoagulants	2	7
Selective serotonin reuptake inhibitors	2	7
Vitamin D supplements	6	20
Number of New Drug Therapies (Incl. Vitamin D)		
0	12	40
1	9	30
2	7	23
3	2	7

*ACE inhibitors, β-blockers or diuretics

As shown in Table 2, most implants were placed in the maxilla (78%) and in posterior sites (76%). Timing of placement was heterogeneous, with 24% placed immediately after extraction (immediate implants), 35% in the early phase, and 41% in a delayed protocol. The most frequent diameter was 4.25 mm (51%), followed by 3.8 mm (35%) and 5.0 mm (14%). Implant length was predominantly 10.0 mm (57%), with 43% measuring 11.5 mm. Gingival phenotype was evenly distributed, with 54% of implants associated with a thin phenotype and 46% with a thick phenotype.

**Table 2:** Clinical parameters of the *n* = 37 Implants with evaluable Marginal Bone Loss (MBL) or Pink Esthetic Score (PES). Data at Ten Years

	*n*	%
*Preoperative Period*		
Location		
Maxilla	29	78
Mandible	8	22
Position		
Anterior	9	24
Posterior	28	76
Timing		
Immediate	9	24
Early	13	35
Delayed	15	41
*Intraoperative Period*		
Diameter, mm		
3.8	13	35
4.25	19	51
5.0	5	14
Length, mm		
10.0	21	57
11.5	16	43
*Postoperative Period*		
Gingival phenotype		
Thin	20	54
Thick	17	46

All implants were still in place, yielding a survival rate of 100% between six and ten years. Success was also 100%, as MBL remained below 2 mm in all cases and no bleeding on probing or suppuration was observed during follow-up. Abutment loosening between 6 and 10 years from loading was described in 3 implants during follow-up (8.1%). All these cases were easily managed as the implant abutment connection was located distant from the bone tissue due to the 2.8 mm convergent neck.

### Onset of systemic disease during the follow up

Ten patients (33%) developed one new systemic disease and two patients (7%) developed two systemic diseases. Eighteen patients (60%) did not develop any new systemic condition. CVDs were the most frequent new diagnoses (27%), whereas oncological, endocrine, autoimmune, metabolic, gastrointestinal disorders and depression were each reported in only one patient (3%). Regarding pharmacological treatments, antihypertensive agents were the most common (33%), followed by corticosteroids and proton pump inhibitors (10% each). All other therapies, including immunological agents, anticoagulants and SSRIs, were reported in limited cases (≤ 7%). Vitamin D supplementation was recorded in 20% of patients.

### Hard tissue variations (MBL)

MBL values are summarized in Table 3. At six years, MBL was 0.88 mm (95% CI, 0.73 to 1.03), and at ten years it remained essentially stable [0.90 mm (95% CI, 0.68 to 1.12); mean change + 0.02 mm (95% CI, − 0.09 to + 0.13), *p* = 0.714]. When stratified by age, patients younger than 60 years showed mean values of 0.81 mm (95% CI, 0.60 to 1.02) at six years and 0.80 mm (95% CI, 0.50 to 1.10) at ten years, while those aged 60 years or older had corresponding values of 1.02 mm (95% CI, 0.85 to 1.18) and 1.09 mm (95% CI, 0.84 to 1.33). Differences between age groups were not statistically significant at either follow-up [mean difference + 0.29 mm (95% CI, − 0.10 to + 0.68), *p* = 0.136 at ten years], as well as between temporal trends [interaction + 0.08 mm (95% CI, − 0.12 to + 0.29), *p* = 0.423]. Similarly, no significant differences were found when comparing patients with and without CVDs [mean difference + 0.26 mm (95% CI, − 0.10 to + 0.62), *p* = 0.151 at ten years] or those under antihypertensive therapy versus not [mean difference + 0.24 mm (95% CI, − 0.14 to + 0.63), *p* = 0.202 at ten years].

**Table 3:** Marginal Bone Loss (MBL, mm) at Six and Ten Years According to Age Group, Cardiovascular Diseases, and Antihypertensive Agents, With Mean Changes Over Time and Between-Group Comparisons

	Six Years	Ten Years	Ten vs. Six Years	
			Mean Change	*P*-Value
All	0.88 (0.73, 1.03)	0.90 (0.68, 1.12)	+ 0.02 (− 0.09, + 0.13)	0.714
Age Group, y				
<60	0.81 (0.60, 1.02)	0.80 (0.50, 1.10)	−0.01 (− 0.16, + 0.14)	0.891
≥60	1.02 (0.85, 1.18)	1.09 (0.84, 1.33)	+ 0.07 (− 0.07, + 0.21)	0.309
≥60 vs. < 60				
Mean Diff.	+ 0.21 (− 0.06, + 0.47)	+ 0.29 (− 0.10, + 0.68)	+ 0.08 (− 0.12, + 0.29)	
* P*-Value	0.119	0.136	0.423	
CVDs				
No	0.87 (0.69, 1.05)	0.86 (0.60, 1.11)	−0.01 (− 0.13, + 0.10)	0.818
Yes	0.95 (0.79, 1.11)	1.12 (0.86, 1.37)	+ 0.17 (− 0.07, + 0.40)	0.157
Yes vs. No				
Mean Diff.	+ 0.08 (− 0.16, + 0.32)	+ 0.26 (− 0.10, + 0.62)	+ 0.18 (− 0.08, + 0.44)	
* P*-Value	0.499	0.151	0.171	
Antihypertensives				
No	0.87 (0.68, 1.06)	0.84 (0.58, 1,11)	−0.02 (− 0.15, + 0.10)	0.690
Yes	0.93 (0.74, 1.12)	1.09 (0.81, 1.36)	+ 0.16 (− 0.03, + 0.34)	0.092
Yes vs. No				
Mean Diff.	+ 0.06 (− 0.20, + 0.33)	+ 0.24 (− 0.14, + 0.63)	+ 0.18 (− 0.04, + 0.40)	
* P*-Value	0.635	0.202	0.105	

All values are means with 95% confidence intervals shown in parentheses. MBL values at six and ten years are available for 33 out of 37 implants

Abbreviations: *CVDs* Cardiovascular Diseases

### Soft tissue variations (PES)

PES values are summarized in Table 4. At six years, the mean PES was 11.9 (95% CI, 11.6 to 12.3), and at ten years it decreased significantly to 11.1 (95% CI, 10.6 to 11.6) [mean change − 0.8 (95% CI, − 1.1 to − 0.5), *p* < 0.001]. When stratified by age, patients younger than 60 years showed mean values of 12.3 (95% CI, 11.9 to 12.7) at six years and 11.6 (95% CI, 11.1 to 12.2) at ten years [mean change − 0.6 (95% CI, − 1.0 to − 0.2), *p* = 0.004]. In contrast, patients aged 60 years or older started from lower values [11.4 (95% CI, 10.8 to 11.9) at six years] and declined further to 10.3 (95% CI, 9.6 to 10.9) at ten years [mean change − 1.1 (95% CI, − 1.6 to − 0.6), *p* < 0.001]. Between-group comparisons confirmed significantly lower PES values in the older group both at six years [mean difference − 0.9 (95% CI, − 1.6 to − 0.2), *p* = 0.009] and at ten years [− 1.4 (95% CI, − 2.2 to − 0.5), *p* = 0.003]. However, temporal trends were not significantly different between groups [interaction − 0.5 (95% CI, − 1.1 to + 0.2), *p* = 0.165]. Similar findings were observed when stratifying by CVDs: patients with CVDs consistently showed lower PES values both at six years [mean difference − 1.1 (95% CI, − 1.9 to − 0.3), *p* = 0.011] and at ten years [− 1.4 (95% CI, − 2.3 to − 0.4), *p* = 0.007], while temporal changes were not significantly different [interaction − 0.3 (95% CI, − 1.1 to + 0.6), *p* = 0.515]. These findings were confirmed in models additionally adjusted for age, with both age and cardiovascular diseases remaining independently associated with lower PES values (data not shown). In contrast, no significant differences were found according to antihypertensive therapy at either follow-up or in temporal trends (all *p* ≥ 0.061).

**Table 4:** Pink Esthetic Score (PES) at Six and Ten Years According to Age Group, Cardiovascular Diseases, and Antihypertensive Agents, With Mean Changes Over Time and Between-Group Comparisons

	Six Years	Ten Years	Ten vs. Six Years	
			Mean Change	*P*-Value
All	11.9 (11.6, 12.3)	11.1 (10.6, 11.6)	−0.8 (− 1.1, − 0.5)	< 0.001*
Age Group, y				
<60	12.3 (11.9, 12.7)	11.6 (11.1, 12.2)	−0.6 (− 1.0, − 0.2)	0.004*
≥60	11.4 (10.8, 11.9)	10.3 (9.6, 10.9)	−1.1 (− 1.6, − 0.6)	< 0.001*
≥60 vs. < 60				
Mean Diff.	−0.9 (− 1.6, − 0.2)	−1.4 (− 2.2, − 0.5)	−0.5 (− 1.1, + 0.2)	
* P*-Value	0.009*	0.003*	0.165	
CVDs				
No	12.2 (11.8, 12.6)	11.5 (11.0, 12.0)	−0.7 (− 1.1, − 0.4)	< 0.001*
Yes	11.1 (10.4, 11.8)	10.1 (9.3, 10.9)	−1.0 (− 1.8, − 0.2)	0.012*
Yes vs. No				
Mean Diff.	−1.1 (− 1.9, − 0.3)	−1.4 (− 2.3, − 0.4)	−0.3 (− 1.1, + 0.6)	
* P*-Value	0.011*	0.007*	0.515	
Antihypertensives				
No	12.2 (11.8, 12.6)	11.4 (10.9, 11.9)	−0.8 (− 1.2, − 0.4)	< 0.001*
Yes	11.4 (10.7, 12.1)	10.6 (9.7, 11.5)	−0.8 (− 1.5, − 0.1)	0.021*
Yes vs. No				
Mean Diff.	−0.8 (− 1.6, + 0.0)	−0.8 (− 1.9, + 0.3)	0.0 (− 0.8, + 0.81)	
* P*-Value	0.061	0.140	1.000	

*p ≤ 0.05

All values are means with 95% confidence intervals shown in parentheses. PES values at six and ten years are available for 30 out of 37 implants

Abbreviations: *CVDs* Cardiovascular Diseases

## Discussion

The study revealed that hard and soft tissues remained stable between six and ten years around the convergent neck despite aging and onset of new pathologies in a substantial proportion of patients. Implant survival rates at 10 years were above 90% for different type of implant systems [21–24].

Implant-related operative parameters such as diameter, insertion timing, location, soft tissue biotype, and implant length were extensively evaluated in the previous 6-year report of this cohort [11], where their association with short- and medium-term outcomes was specifically analyzed. The present 10-year investigation was not designed to re-examine these baseline surgical and prosthetic variables, but rather to assess long-term biological stability between six and ten years and to explore the potential impact of aging and newly occurring systemic conditions on peri-implant tissues.

Interestingly, no significant differences were observed suggesting a minor role of these conditions after a long period of occlusal load and a recurrent hygiene re-call procedure.

The placement of implant at transmucosal level created the condition for the formation of a gingival tunnel that resulted undisturbed by crown design and morphology. As well visible in radiographs, all finishing crown margin was gently in contact with gingival tissue and did not invade the gingival tunnel. It is probable that internal mucosa of gingival tunnel remained in close contact with the microtextured surface of collar neck and created connective fibers to support and preserve a stable sealing and an adequate vascularization to perform bone stability.

Our data revealed that MBL remained stable between the 6-year and 10-year follow-up assessments. The stable hard tissues (mean MBL 0.90 mm; 95%CI 0.68–1.12) supports the rationale of the ZirTi surface treatment and the convergent transmucosal microtextured collar, designed to minimize marginal bone remodeling and to preserve peri-implant mucosa [25, 26]. Histological studies support of the hard tissue response around this ZirTi implant surface, reporting high bone implant contact in between the threads [12, 27]. Clinical studies found minimal bone loss during the first year of load when this implant is placed with the neck partially exposed [10, 28]. A systematic review and meta-analysis also confirm that implant neck convergent profile resulted in a more stable MBL [29]. No other studies with a 10-year follow-up are reported regarding Prama implant.

The soft tissue response to the convergent neck was favorable in previous studies [10, 28]. Clinically, the transmucosal implant supported papilla growth and coronal soft tissue thickening during the first 1–5 years of loading. Morón-Conejo et al. found greater horizontal gingival thickening with PRAMA than with Premium implants, though not statistically significant [28]. Histological studies on this implant confirm that the microtextured titanium collar (Ultrathin Threaded Microsurface, UTM, ~ 60 μm microgrooves) promotes collagen fiber adhesion and healthy mucosal integration. Animal models show connective tissue fibers oriented obliquely or perpendicularly to the collar, resembling the attachment around natural teeth [12].

PES was selected as assessment tool for soft tissue response around implant. The analysis of the PES variables (mesial papilla, distal papilla, soft tissue level, soft tissue contour, alveolar process deficiency, soft tissue color, and soft tissue texture), allows to detect qualitative changes in the peri-implant mucosa, including variations that may reflect altered tissue conditions.

Interestingly, at 10 years, PES exhibited some variations to previous follow-ups. Mean PES at showed a slight decrease when compared to 6-year data (from 11.9 (95%CI 11.6 to 12.3) to 11.1 (95%CI 10.6 to 11.6). Such findings highlight the dynamic nature of the peri-implant mucosa, which may continue to remodel even in the absence of radiographical bone loss or implant related inflammatory events.

All metal ceramic crowns were applied 10 years ago and were designed with traditional impressions procedures and following cementation. The absence of crown compression and the easy cleanability of the restorations are due to the BOPT principles applied, particularly the hyperbolic neck design of Prama and feather-edge preparation [30]. This concept seems to promote soft-tissue adaptation and reduces mechanical stresses at the implant–abutment interface, all factors contributing to long-term biological and mechanical stability [31]. Only a limited number of prosthetic complications were observed, mainly represented by abutment loosening (8.1%) that suggest the validity of the present prosthetic protocol.

Some additional aspects must be considered in relation of the present cohort. Baseline patient age was approx. 56 years, meaning that after 10 years, a greater percentage of patients may be considered in the elderly age (approx. 75% of patients) [32]. This aspect must be considered as new systemic pathologies or new pharmacological regimens could critically affect the peri implant tissues and exacerbate peri-implant inflammation or bone loss despite initially favorable clinical performance [33, 34].

We report the significant relation of two non-implant related factors, namely aging and occurrence of new CVD on PES at 10 years (*p* < 0.050). Changes in mucosal thickness and contour reflect the physiological adaptation and aging of peri-implant soft tissues rather than pathological causes. A recent study reported that oral mucosa undergoes structural dehydration and collagen destabilization with age and that patients with cardiovascular diseases exhibit marked and altered collagen reorganization [35].

Despite the long-term evaluation and the appearance of new system diseases in an important part of the cohort population, no significant modification was observed in MBL and PES parameter. Further evaluation must be considered and interpreted considering the age of the study cohort. In the present study, no PES scores of 8 or less were observed, a threshold considered clinically unacceptable [13, 36].

Limitations of this study include the considerable drop-out of the initially enrolled cohort, which appears comparable to that reported in previous long-term implant investigations. Attrition over the 10-year follow-up may have influenced survival and success estimates. Moreover, longitudinal analyses were restricted to implants available at the six-year follow-up and re-evaluated at ten years. Therefore, survival, success, and longitudinal change estimates should be interpreted as conditional on continued participation and implant presence at mid-term follow-up. Other limitations include the strict intervention protocol and flapless surgery performed by an experienced operator, which may have influenced the final outcome.

## Conclusions

Within the limitations of available data, transmucosal implants with a convergent neck showed stable marginal bone levels over a 10-year follow-up. The cohort population was affected by new pathologies in an aging population, but did not result in significant clinical parameter (PES) modification. The negative effect of systemic diseases and aging must be considered in future long-term study.

The proposed protocol, preventing any invasion of the gingival tunnel and preservation of soft tissues contour in association with the transmucosal approach may have contributed to the stability of the tissues.

## Data Availability

Data available upon reasonable requests.
